# Supplementary material for: Pregnancy complications and maternal birth outcomes in women with intellectual and developmental disabilities in Wisconsin Medicaid
Source: PLoS One. 2020 Oct 27;15(10):e0241298. doi: 10.1371/journal.pone.0241298 (PMC7591078; doi:10.1371/journal.pone.0241298)
Supplement: S1 Table — (DOCX) [file pone.0241298.s001.docx]

S1 Table. International classification of disease 9 and 10 codes used to identify intellectual and developmental disabilities

| **Condition** | **ICD 9 Code** | **IDD 10 code** | | |
| --- | --- | --- | --- | --- |
| **Autism Spectrum disorder** (including pervasive developmental disorders, Asperger's syndrome | 299 | F840 |  | |
|  | 29900 | F841 |  | |
|  | 29901 | F845 |  | |
|  | 29980 | F848 |  | |
|  | 29981 | F849 |  | |
|  | 29990 |  |  | |
|  |  |  |  | |
| **Intellectual disability** (mild, moderate, severe, profound, other, unspecified | 317 | F700 | F729 | |
|  | 3180 | F701 | F730 | |
|  | 3181 | F708 | F731 | |
|  | 3182 | F709 | F738 | |
|  | 319 | F710 | F739 | |
|  |  | F711 | F780 | |
|  |  | F718 | F781 | |
|  |  | F719 | F788 | |
|  |  | F720 | F789 | |
|  |  | F721 | F790 | |
|  |  | F728 | F791 | |
|  |  | F799 | F798 | |
|  |  |  |  | |
| **Cerebral palsy** (athetoid, diplegic, hemiplegic, quadriplegic, other, infantile, spastic) | 33371 | G80 |  | |
|  | 3430 | G800 |  | |
|  | 3431 | G801 |  | |
|  | 3432 | G802 |  | |
|  | 3433 | G803 |  | |
|  | 3434 | G804 |  | |
|  | 3438 | G808 |  | |
|  | 3439 | G809 |  | |
|  | 34389 |  |  | |
|  |  |  |  | |
| **Genetic conditions** |  |  |  | |
| Chromosomal anomalies | 758, 7598 | Q99.8 |  | |
| Down's syndrome | 7580 | Q90.0, Q90.1, | | Q90.2, Q90.9 |
| Patau's syndrome | 7581 | Q91.4, Q91.5, | | Q91.6, Q91.7 |
| Edwards' syndrome | 7582 | Q91.0, Q91.1, | | Q91.2, Q91.3 |
| Autosomal deletion syndromes | 7583 |  |  | |
| Cri-du-chat syndrome | 75831 | Q93.4 |  | |
| Velo-cardio-facial syndrome | 75832 | Q93.81 |  | |
| Other microdeletions | 75833 | Q93.88 |  | |
| Other autosomal deletions | 75839 | Q93.51, Q93.59 | | Q93.82, Q93.8 |
| Tuberous sclerosis | 7595 | Q851 |  | |
| Prader Wili | 75981 | Q871 |  | |
| Fragile X | 75983 | Q99.2 |  | |
| Rubinstein/Taybi | 75989 | Q8723 |  | |
| Lesch Nyhand | 2772 | E791 |  | |
| Rett's Syndrome | 3308 | F842 |  | |
| Sotos | 253 | Q8731 |  | |
|  |  |  |  | |
| **Other** |  |  |  | |
| Child disintegrative disorder | 29910 | F843, | | F844 |
| Fetal Alcohol/ hydantoin syndrome | 76071, 76077 | Q860, | | Q861 |
| Developmental disability, NOS | 3157 |  |  | |
| Other congenital abnormalities NOS | 7598 | Q878 |  | |
