# Supplementary material for: Pregnancy complications and maternal birth outcomes in women with intellectual and developmental disabilities in Wisconsin Medicaid
Source: PLoS One. 2020 Oct 27;15(10):e0241298. doi: 10.1371/journal.pone.0241298 (PMC7591078; doi:10.1371/journal.pone.0241298)
Supplement: S2 Table — (DOCX) [file pone.0241298.s002.docx]

| S2 Table. Demographic characteristics of mothers with a live Medicaid covered birth in Wisconsin, 2007-2016; by Intellectual and developmental disability type | | | | | | | | |
| --- | --- | --- | --- | --- | --- | --- | --- | --- |
|  | Intellectual disability | | Genetic condition | | Cerebral Palsy | | Autism | |
|  | N=331 | | N=436 | | N=170 | | N=102 | |
|  | N | % | N | % | N | % | N | % |
| **Maternal race ethnicity** | |  |  |  |  |  |  |  |
| White | 193 | 58.3 | 334 | 76.6 | 135 | 79.4 | 83 | 81.4 |
| Black | 112 | 33.8 | 72 | 16.5 | 28 | 16.5 | 14 | 13.7 |
| Other | 26 | 7.9 | 30 | 6.9 | - | - | - | - |
|  |  |  |  |  |  |  |  |  |
| Hispanic | 27 | 7.6 | 60 | 13.8 | 17 | 9.4 | - | - |
| Not Hispanic | 304 | 92.4 | 376 | 86.2 | 153 | 90.6 | - | - |
|  |  |  |  |  |  |  |  |  |
| **Marital status** |  |  |  |  |  |  |  |  |
| Yes | 45 | 13.6 | 150 | 34.4 | 64 | 37.7 | 28 | 27.5 |
| No | 286 | 86.7 | 286 | 65.6 | 106 | 62.4 | 74 | 72.5 |
|  |  |  |  |  |  |  |  |  |
| **Maternal education** |  |  |  |  |  |  |  |  |
| <High school | 117 | 36.1 | 91 | 20.9 | 38 | 22.4 | 24 | 22.0 |
| Completed high school | 185 | 57.1 | 182 | 41.7 | 88 | 51.8 | 51 | 51.0 |
| >=Some college | 21 | 6.5 | 158 | 36.2 | 42 | 24.7 | 27 | 27.0 |
|  |  |  |  |  |  |  |  |  |
| **# of live births over 11 years** | | |  |  |  |  |  |  |
| 1 | 191 | 57.7 | 205 | 47.0 | 100 | 58.8 | 65 | 63.7 |
| 2 | 87 | 26.3 | 148 | 33.9 | 42 | 24.7 | 25 | 24.5 |
| 3+ | 52 | 15.7 | 83 | 19.0 | 28 | 16.5 | 12 | 11.8 |
|  |  |  |  |  |  |  |  |  |
| **Maternal age at birth** |  |  |  |  |  |  |  |  |
| Mean, SD | 25.3 | 6.0 | 27.3 | 6.0 | 25.9 | 5.3 | 26.0 | 5.8 |
| Median, IQR | 24.0 | 7.9 | 27.0 | 8.3 | 25.2 | 7.1 | 25.5 | 8.9 |
|  |  |  |  |  |  |  |  |  |
|  |  |  |  |  |  |  |  |  |
| Demographic taken from random sampling of birth records if mother had more than one birth during the period | | | | | | | | |
