# Supplementary material for: Pregnancy complications and maternal birth outcomes in women with intellectual and developmental disabilities in Wisconsin Medicaid
Source: PLoS One. 2020 Oct 27;15(10):e0241298. doi: 10.1371/journal.pone.0241298 (PMC7591078; doi:10.1371/journal.pone.0241298)
Supplement: S3 Table — (DOCX) [file pone.0241298.s003.docx]

| S3 Table. Demographic characteristics of mothers with live birth in Wisconsin 2007-2016 comparing intellectual and developmental disability identiifcation criteria in Medicaid claims one-year pre pregnancy | | | | | | | | | |
| --- | --- | --- | --- | --- | --- | --- | --- | --- | --- |
|  | Intellectual and developmental disabilities, 1 claim, at last birth | |  | Intellectual and developmental disabilities, 2 claim, at last birth | |  | Intellectual and developmental disabilities, 2 claim, 2 different days, at last birth | |  |
|  | **By mother** | | | | | | | | |
|  | N=1032 | |  | N=986 | |  | N=830 | |  |
|  | N | % |  | N | % |  | N | % |  |
|  |  |  |  |  |  |  |  |  |  |
| **Maternal race** |  |  |  |  |  |  |  |  |  |
| White | 736 | 71.3 |  | 702 | 71.2 |  | 588 | 70.8 |  |
| Black | 223 | 21.6 |  | 211 | 21.4 |  | 181 | 21.8 |  |
| Asian | 49 | 4.7 |  | 49 | 5.0 |  | 43 | 5.2 |  |
| Other | 24 | 2.3 |  | 21 | 2.1 |  | 18 | 2.2 |  |
|  |  |  |  |  |  |  |  |  |  |
| **Hispanic ethnicity** |  |  |  |  |  |  |  |  |  |
| Hispanic | 107 | 10.4 |  | 103 | 10.4 |  | 80 | 9.6 |  |
| Non-Hispanic | 925 | 89.6 |  | 883 | 89.6 |  | 750 | 90.4 |  |
|  |  |  |  |  |  |  |  |  |  |
| **Mother foreign born** | |  |  |  |  |  |  |  |  |
| Yes | 56 | 5.4 |  | 54 | 5.5 |  | 37 | 4.5 |  |
| No | 976 | 94.6 |  | 932 | 94.5 |  | 798 | 96.1 |  |
| Missing |  |  |  |  |  |  |  |  |  |
|  |  |  |  |  |  |  |  |  |  |
| **Maternal education** | |  |  |  |  |  |  |  |  |
| <High school | 254 | 24.8 |  | 245 | 25.1 |  | 200 | 24.3 |  |
| Completed high school | 499 | 48.7 |  | 482 | 49.3 |  | 419 | 51.0 |  |
| Some college | 218 | 21.3 |  | 198 | 20.2 |  | 161 | 19.6 |  |
| ≥ Completed college | 53 | 5.2 |  | 53 | 5.4 |  | 42 | 5.1 |  |
| Missing | - |  |  | - |  |  | - |  |  |
|  |  |  |  |  |  |  |  |  |  |
| **Marital status** |  |  |  |  |  |  |  |  |  |
| Married | 298 | 28.9 |  | 284 | 26.8 |  | 231 | 27.8 |  |
| Not Married | 734 | 71.1 |  | 702 | 71.2 |  | 599 | 72.2 |  |
| Missing |  |  |  |  |  |  |  |  |  |
|  |  |  |  |  |  |  |  |  |  |
| **Number of births in BD4LK** | |  |  |  |  |  |  |  |  |
| 1 | 560 | 54.3 |  | 542 | 55.0 |  | 464 | 55.9 |  |
| 2 | 293 | 28.4 |  | 278 | 28.2 |  | 228 | 27.5 |  |
| 3+ | 179 | 17.3 |  | 166 | 16.8 |  | 138 | 16.6 |  |
|  | **By births** | | | | | | | | |
|  | N=1757 | |  | N=1665 | |  | N=1389 | |  |
|  | N | % |  | N | % |  | N | % |  |
| **Maternal age at birth** |  |  |  |  |  |  |  |  |  |
| ≤18 | 33 | 4.3 |  | 68 | 4.1 |  | 56 | 4.0 |  |
| 19-24 | 304 | 39.6 |  | 728 | 43.7 |  | 591 | 42.6 |  |
| 25-29 | 235 | 30.6 |  | 457 | 27.4 |  | 394 | 28.4 |  |
| 30-34 | 121 | 15.8 |  | 256 | 15.4 |  | 214 | 15.4 |  |
| 35-39 | 58 | 7.6 |  | 116 | 7.0 |  | 99 | 7.1 |  |
| ≥40 | 16 | 2.1 |  | 41 | 2.5 |  | 33 | 2.4 |  |
| Mean age, SD | 26.5 | 6.0 |  | 26.3 | 6.0 |  | 26.4 | 6.0 |  |
| Median age, IQR | 26.0 | 8.3 |  | 25.4 | 8.2 |  | 25.5 | 8.3 |  |
|  |  |  |  |  |  |  |  |  |  |
| **Year of birth** |  |  |  |  |  |  |  |  |  |
| 2007 | 180 | 10.2 |  | 169 | 10.2 |  | 154 | 11.1 |  |
| 2008 | 243 | 13.8 |  | 236 | 14.2 |  | 194 | 14.0 |  |
| 2009 | 222 | 12.6 |  | 209 | 12.6 |  | 181 | 13.0 |  |
| 2010 | 192 | 10.9 |  | 183 | 11.0 |  | 155 | 11.2 |  |
| 2011 | 195 | 11.1 |  | 187 | 11.2 |  | 155 | 11.2 |  |
| 2012 | 193 | 11.0 |  | 187 | 11.2 |  | 154 | 11.1 |  |
| 2013 | 136 | 7.7 |  | 129 | 7.7 |  | 109 | 7.8 |  |
| 2014 | 149 | 8.5 |  | 141 | 8.5 |  | 113 | 8.1 |  |
| 2015 | 127 | 7.2 |  | 117 | 7.0 |  | 97 | 7.0 |  |
| 2016 | 120 | 6.8 |  | 107 | 6.4 |  | 77 | 5.5 |  |
|  |  |  |  |  |  |  |  |  |  |
| **Parity** |  |  |  |  |  |  |  |  |  |
| First born | 557 | 31.7 |  | 534 | 32.1 |  | 462 | 33.3 |  |
| Second born | 497 | 28.3 |  | 468 | 28.1 |  | 387 | 27.9 |  |
| Third born | 334 | 19.0 |  | 311 | 18.7 |  | 249 | 17.9 |  |
| Fourth born or later | 369 | 21.0 |  | 353 | 21.2 |  | 289 | 20.8 |  |
| Missing |  |  |  |  |  |  |  |  |  |
|  |  |  |  |  |  |  |  |  |  |
| **Multiple birth** |  |  |  |  |  |  |  |  |  |
| Yes | 26 | 1.5 |  | 26 | 1.6 |  | 20 | 1.5 |  |
| No | 1696 | 98.5 |  | 1607 | 98.4 |  | 1342 | 98.5 |  |
| Missing |  |  |  |  |  |  |  |  |  |
|  |  |  |  |  |  |  |  |  |  |
| **County type at birth** | |  |  |  |  |  |  |  |  |
| Large central metro | 546 | 31.1 |  | 518 | 31.1 |  | 425 | 30.6 |  |
| Large fringe metro | 135 | 7.7 |  | 139 | 8.3 |  | 106 | 7.6 |  |
| Medium metro | 247 | 14.1 |  | 218 | 13.1 |  | 178 | 12.8 |  |
| Small metro | 448 | 25.5 |  | 438 | 26.3 |  | 366 | 26.3 |  |
| Micropolitan | 195 | 11.1 |  | 183 | 11.0 |  | 160 | 11.5 |  |
| Non-core | 186 | 10.6 |  | 179 | 10.8 |  | 152 | 10.9 |  |
|  |  |  |  |  |  |  |  |  |  |
