# Supplementary material for: Pregnancy complications and maternal birth outcomes in women with intellectual and developmental disabilities in Wisconsin Medicaid
Source: PLoS One. 2020 Oct 27;15(10):e0241298. doi: 10.1371/journal.pone.0241298 (PMC7591078; doi:10.1371/journal.pone.0241298)
Supplement: S4 Table — (DOCX) [file pone.0241298.s004.docx]

| S4 Table. Occurrence and risk ratios of maternal pregnancy complications and adverse outcomes for all births to mothers with intellectual and developmental disabilities compared to the full Wisconsin Medicaid sample of mothers, 2007-2016 with sensitvity analyses for IDD claim count and excludinig years | | | | | | | | | | | | | | | | | |
| --- | --- | --- | --- | --- | --- | --- | --- | --- | --- | --- | --- | --- | --- | --- | --- | --- | --- |
|  | Mothers with Intellectual and developmental disabilities | |  | All mothers in Medicaid | |  | Regression models | | | | |  | Sensitvity analyses | | | | |
|  | N=1757 | |  | N=274865 | |  | Unadjusted risk ratio^a^ | |  | Adjusted risk ratio^b^ | |  | 2 IDD claims | |  | No years 2007, 2013, 2014 | |
|  | N | % |  | N | % |  | RR | 95% CI |  | RR | 95% CI |  | URR | 95% CI |  | URR | 95% CI |
| **Service use** |  |  |  |  |  |  |  |  |  |  |  |  |  |  |  |  |  |
| **Trimester prenatal care began** | |  |  |  |  |  |  |  |  |  |  |  |  |  |  |  |  |
| 1 | 1240 | 72.8 |  | 195789 | 73.4 |  | 0.99 | 0.9, 1.0 |  | 0.99 | 0.9, 1.0 |  | 0.99 | 0.9, 1.0 |  | 0.99 | 0.9, 1.0 |
| 2 | 364 | 21.4 |  | 57708 | 21.6 |  |  |  |  |  |  |  |  |  |  |  |  |
| 3 | 71 | 4.2 |  | 10984 | 4.1 |  |  |  |  |  |  |  |  |  |  |  |  |
| None | 28 | 1.6 |  | 2261 | 0.8 |  | **1.83** | 1.2, 2.8 |  | - | - |  |  |  |  |  |  |
| Missing | 54 |  |  | 8123 |  |  |  |  |  |  |  |  |  |  |  |  |  |
|  |  |  |  |  |  |  |  |  |  |  |  |  |  |  |  |  |  |
| **WIC^c^** |  |  |  |  |  |  |  |  |  |  |  |  |  |  |  |  |  |
| Yes | 712 | 79.6 |  | 102610 | 66.6 |  | **1.20** | 1.1, 1.3 |  | **1.11** | 1.0,1.2 |  | 1.21 | 1.1, 1.3 |  | **1.19** | 1.1, 1.2 |
| No | 183 | 20.4 |  | 51452 | 33.4 |  |  |  |  |  |  |  |  |  |  |  |  |
| Missing | 25 |  |  | 31364 |  |  |  |  |  |  |  |  |  |  |  |  |  |
|  |  |  |  |  |  |  |  |  |  |  |  |  |  |  |  |  |  |
| **Complications and adverse outcomes of pregnancy** | | |  |  |  |  |  |  |  |  |  |  |  |  |  |  |  |
| **Prepregnancy BMI^c^** |  |  |  |  |  |  |  |  |  |  |  |  |  |  |  |  |  |
| Underweight^d^ | 41 | 4.6 |  | 4768 | 3.1 |  | **1.67** | 1.2, 2.3 |  | - | - |  |  |  |  |  |  |
| Normal weight | 277 | 30.9 |  | 57222 | 37.2 |  |  |  |  |  |  |  |  |  |  |  |  |
| Overweight | 220 | 24.5 |  | 40044 | 26 |  |  |  |  |  |  |  |  |  |  |  |  |
| Obese^d^ | 360 | 40.1 |  | 51807 | 33.7 |  | **1.22** | 1.1, 1.3 |  | **1.12** | 1.0, 1.3 |  | 1.23 | 1.1, 1.3 |  | **1.24** | 1.1, 1.4 |
| Missing | 22 |  |  | 31585 |  |  |  |  |  |  |  |  |  |  |  |  |  |
|  |  |  |  |  |  |  |  |  |  |  |  |  |  |  |  |  |  |
| **Ever smoke during pregnancy** | |  |  |  |  |  |  |  |  |  |  |  |  |  |  |  |  |
| Yes | 521 | 29.9 |  | 72933 | 26.7 |  | **1.09** | 1.0, 1.2 |  | 1.00 | 0.9, 1.1 |  | 1.09 | 1.0, 1.2 |  | 1.10 | 1.0 ,1.2 |
| No | 1220 | 70.1 |  | 200661 | 73.3 |  |  |  |  |  |  |  |  |  |  |  |  |
| Missing | 16 |  |  | 1271 |  |  |  |  |  |  |  |  |  |  |  |  |  |
|  |  |  |  |  |  |  |  |  |  |  |  |  |  |  |  |  |  |
| **Gestational diabetes** |  |  |  |  |  |  |  |  |  |  |  |  |  |  |  |  |  |
| Yes | 123 | 7.0 |  | 15108 | 5.5 |  | **1.28** | 1.0, 1.6 |  | **1.37** | 1.1, 1.7 |  | **1.32** | 1.1, 1.6 |  | **1.28** | 1.0, 1.6 |
| No | 1634 | 93.0 |  | 259757 | 94.5 |  |  |  |  |  |  |  |  |  |  |  |  |
|  |  |  |  |  |  |  |  |  |  |  |  |  |  |  |  |  |  |
| **Gestational hypertension** | |  |  |  |  |  |  |  |  |  |  |  |  |  |  |  |  |
| Yes | 106 | 6.0 |  | 13850 | 5 |  | **1.22** | 1.0 ,1.5 |  | **1.30** | 1.0, 1.7 |  | **1.25** | 1.0, 1.5 |  | **1.40** | 1.1, 1.7 |
| No | 1651 | 94.0 |  | 261015 | 95 |  |  |  |  |  |  |  |  |  |  |  |  |
|  |  |  |  |  |  |  |  |  |  |  |  |  |  |  |  |  |  |
| **Complications of delivery** | | |  |  |  |  |  |  |  |  |  |  |  |  |  |  |  |
| **Maternal transfer** |  |  |  |  |  |  |  |  |  |  |  |  |  |  |  |  |  |
| Yes | 26 | 1.5 |  | 2156 | 0.8 |  | **1.86** | 1.2, 2.8 |  | - | - |  | **1.88** | 1.2, 2.9 |  | **1.77** | 1.1, 2.9 |
| No | 1727 | 98.5 |  | 272396 | 99.2 |  |  |  |  |  |  |  |  |  |  |  |  |
| Missing | - |  |  | 313 |  |  |  |  |  |  |  |  |  |  |  |  |  |
|  |  |  |  |  |  |  |  |  |  |  |  |  |  |  |  |  |  |
| **Prolonged labor** |  |  |  |  |  |  |  |  |  |  |  |  |  |  |  |  |  |
| Yes | 19 | 1.1 |  | 3153 | 1.1 |  | 0.95 | 0.6, 1.5 |  | - | - |  | 0.95 | 0.6,1.5 |  | 0.80 | 0.4, 1.5 |
| No | 1735 | 98.9 |  | 271412 | 98.9 |  |  |  |  |  |  |  |  |  |  |  |  |
| Missing | - |  |  | 300 |  |  |  |  |  |  |  |  |  |  |  |  |  |
|  |  |  |  |  |  |  |  |  |  |  |  |  |  |  |  |  |  |
| **Precipitous labor** |  |  |  |  |  |  |  |  |  |  |  |  |  |  |  |  |  |
| Yes | 85 | 4.8 |  | 11613 | 4.2 |  | 1.17 | 0.9, 1.4 |  | - | - |  | 1.20 | 0.9, 1.5 |  | 1.12 | 0.9, 1.5 |
| No | 1669 | 95.2 |  | 262952 | 95.8 |  |  |  |  |  |  |  |  |  |  |  |  |
| Missing | - |  |  | 300 |  |  |  |  |  |  |  |  |  |  |  |  |  |
|  |  |  |  |  |  |  |  |  |  |  |  |  |  |  |  |  |  |
| **Induced labor** |  |  |  |  |  |  |  |  |  |  |  |  |  |  |  |  |  |
| Yes | 498 | 28.4 |  | 75461 | 27.5 |  | 1.02 | 0.9, 1.1 |  | 1.02 | 0.9, 1.1 |  | 1.00 | 0.9, 1.1 |  | 1.03 | 0.9, 1.1 |
| No | 1254 | 71.6 |  | 199179 | 72.5 |  |  |  |  |  |  |  |  |  |  |  |  |
| Missing | - |  |  | 225 |  |  |  |  |  |  |  |  |  |  |  |  |  |
|  |  |  |  |  |  |  |  |  |  |  |  |  |  |  |  |  |  |
| **Caesarean delivery** |  |  |  |  |  |  |  |  |  |  |  |  |  |  |  |  |  |
| Yes | 436 | 26.3 |  | 54802 | 20.9 |  | **1.3** | 1.2, 1.4 |  | **1.33** | 1.2, 1.5 |  | 1.31 | 1.2, 1.4 |  | 1.31 | 1.2, 1.5 |
| No | 1220 | 73.7 |  | 207828 | 79.1 |  |  |  |  |  |  |  |  |  |  |  |  |
| Missing | 101 |  |  | 12134 |  |  |  |  |  |  |  |  |  |  |  |  |  |
