# Supplementary material for: Pregnancy complications and maternal birth outcomes in women with intellectual and developmental disabilities in Wisconsin Medicaid
Source: PLoS One. 2020 Oct 27;15(10):e0241298. doi: 10.1371/journal.pone.0241298 (PMC7591078; doi:10.1371/journal.pone.0241298)
Supplement: S5 Table — (DOCX) [file pone.0241298.s005.docx]

S5 Table. Analysis of caesarean delivery in women with and without intellectual and developmental disabilities in Wisconsin Medicaid, 2007-2016

|  | Intellectual and developmental disabilities | |  | Total Medicaid population | |  | Unadjusted risk ratio | |
| --- | --- | --- | --- | --- | --- | --- | --- | --- |
|  | N=1757 | |  | N=273108 | |  |  |  |
|  |  |  |  |  |  |  |  |  |
| **Caesarean delivery** |  |  |  |  |  |  |  |  |
| Yes | 436 | 26.3 |  | 54366 | 20.8 |  | **1.32** | 1.2, 1.4 |
| Term | 180 | 33.5 |  | 15719 | 24.0 |  | **1.27** | 1.2, 1.4 |
| Preterm | 357 | 66.5 |  | 49993 | 76.0 |  | **1.25** | 1.1, 1.4 |
|  |  |  |  |  |  |  |  |  |
| Nulliparous | 172 | 39.5 |  | 22178 | 41.1 |  | **1.30** | 1.1, 1.5 |
| Primiparous | 264 | 60.6 |  | 31752 | 58.9 |  | **1.29** | 1.1, 1.5 |
|  |  |  |  |  |  |  |  |  |
| First C-section | 172 | 39.4 |  | 22602 | 41.2 |  | **1.28** | 1.1, 1.5 |
| Previous C section | 264 | 60.6 |  | 31764 | 58.0 |  | 1.04 | 0.9, 1.1 |
|  |  |  |  |  |  |  |  |  |
| Breach | 53 | 12.2 |  | 5986 | 10.9 |  | 1.04 | 0.9, 1.1 |
| Non Breach | 383 | 87.8 |  | 48140 | 87.8 |  | **1.33** | 1.2, 1.5 |
|  |  |  |  |  |  |  |  |  |
| No | 1220 | 73.7 |  | 206608 | 78.6 |  |  |  |
| Missing | 101 |  |  | 12033 |  |  |  |  |
