# Supplementary material for: Pregnancy complications and maternal birth outcomes in women with intellectual and developmental disabilities in Wisconsin Medicaid
Source: PLoS One. 2020 Oct 27;15(10):e0241298. doi: 10.1371/journal.pone.0241298 (PMC7591078; doi:10.1371/journal.pone.0241298)
Supplement: S1 File — (PDF) [file pone.0241298.s006.pdf]

Birth outcomes affecting infants of mothers with intellectual and developmental disabilities

Eric Rubenstein[a], PhD ScM, Deborah B. Ehrenthal[b,c], MD MPH, David Mallinson MS[b], Lauren Bishop,[a,d] PhD MSW, Hsiang-Huo Kuo[c] PhD, Maureen S. Durkin[a,b], DrPH, PhD

Affiliations: [a]Waisman Center, University of Wisconsin-Madison, Madison, Wisconsin
[b] Department of Population Health Science, University of Wisconsin School of Medicine and Pubic Health, Madison, Wisconsin
[c] Department of Obstetrics and Gynecology, University of Wisconsin School of Medicine and Public Health
[d] Department of Social Work, University of Wisconsin-Madison, Madison, Wisconsin

Address correspondence to:
Eric Rubenstein
Waisman Center, University of Wisconsin Madison
T113, 1500 Highland Ave, Madison WI
Erubenstein2@wisc.edu
732-803-2067

Short title: Birth outcomes for children of mothers with IDD

Funding: This work was supported by the Eunice Kennedy Shriver National Institute for Child Health and Human Development (R03HD099619, T32 HD007014-42) and the University of Wisconsin-Madison Clinical and Translational Science Award programme through the National Institutes of Health National Center for Advancing Translational Sciences (UL1TR00427, KL2 TR002374), by the University of Wisconsin-Madison School of Medicine and Public Health's Wisconsin Partnership Program, and by the University of Wisconsin- Madison Institute for Research on Poverty. This study was supported in part by a core grant to the Waisman Center from the *Eunice Kennedy Shriver* National Institute of Child Health and Human Development (U54 HD090256).

Financial disclosure: The authors have indicated they have no financial relationships relevant to this article to disclose

Conflict of interests: The authors have indicated they have no conflicts of interests relevant to this article to disclose

Abbreviations: IDD; intellectual and developmental disabilities, BD4LK; Big Data for Little Kids, NICU; neonatal intensive care unit, OR; odds ratio, uOR; unadjusted odds ratio, aOR; adjusted odds ratio, SD; standard deviation, IQR; interquartile range

Table of contents summary: Wisconsin Medicaid data linked to birth records find infants of mothers with intellectual and developmental disabilities are at higher odds of poor birth outcomes.

What is known on this subject: Infants born to mothers with intellectual and developmental disabilities may be at increased risk of being born preterm and of low birthweight; yet, more work is needed to confirm past findings and understand impacts of demographics and maternal pregnancy complications.

What this study adds: Infants born to mothers with intellectual and developmental disabilities in Wisconsin Medicaid are at increased odds, relative to infants, of being born

preterm, low birthweight and small for gestational age with little attenuation after adjustment for maternal complications and demographic factors.

Dr. Rubenstein conceptualized and designed the study, carried out the analyses, drafted the manuscript, and reviewed and revised the manuscript.

Dr. Ehrenthal led the larger project in which the data were accessed, linked, and cleaned. Dr. Ehrenthal reviewed and revised the manuscript.

Dr. Kuo and Mr. Mallinson contributed to data cleaning, study design, and reviewed and revised the manuscript.

Drs. Bishop and Durkin reviewed and revised the manuscript.

Abstract: 250 words

Objectives: To examine birth outcomes of infants born to mothers with intellectual and developmental disabilities (IDD) and assess effects of demographics, maternal pregnancy complications, and IDD-type.
Methods: We used data from the Big Data for Little Kids Project which links Wisconsin birth records to Medicaid claims for live births covered by Medicaid from 2007 to 2016. We identified IDD using maternal prepregnancy Medicaid claims and ran multi-level logistic regressions clustered by mother to compare outcomes between infants with and without mothers with IDD. We added covariate sets for demographic factors and maternal pregnancy complications to examine confounding and potential mediation. We assessed outcomes by IDD-type (intellectual disability, genetic conditions, cerebral palsy, and autism spectrum disorder) to explore differences by categories of IDD.
Results: Infants of mothers with IDD were at greater odds of being born preterm (odds ratio [OR]: 1.60, 95% CI: 1.4, 1.8), low birth weight (OR: 1.90, 95% CI: 1.6, 2.2), and small for gestational age (OR: 1.5, 95% CI: 1.3, 1.7). Results were robust to adjustment for demographics and did not change when accounting for maternal pregnancy complications. Estimates did not meaningfully differ when grouped by IDD-type.
Conclusions: Infants born to mothers with IDD covered by Medicaid were at elevated odds for poor outcomes, which were not fully attributable to demographic differences of maternal complications. It is imperative to understand why children of mothers with IDD are at elevated risk so interventions and management can be developed to improve outcomes.

Intellectual and developmental disabilities (IDD) are conditions that present before the age of 18 and entail significant limitations in cognition, communication, and adaptive functioning (1). While IDD should not affect a person's reproductive rights, women with IDD face disparity and inequities in reproductive health (2, 3). In addition, infants born to mothers with IDD may be at higher risk of being born preterm (4, 5) and small for gestational age (6) compared to infants of mothers without IDD. Long term child outcomes may be compounded if infants are born into socioeconomically disadvantaged situations, as infants with mothers with IDD often are (7, 8).

While evidence suggests increased risk of adverse outcomes for infants born to mothers with IDD, more work is needed to replicate past findings, assess additional infant outcomes, and examine impact of other contextual factors. US Medicaid data allow us to identify IDD through claims in a state-level system designed to serve low-income people, many of which meet disability eligibility. Medicaid samples may better account for socioeconomic confounding, as women with IDD are compared to a more similar low-income comparison group (9). The effect of maternal complications on infant outcomes in the context of IDD is largely unknown, as mothers with IDD are at greater risk of gestational diabetes and gestational hypertension (10, 11) compared to mothers without IDD. Increased risk for outcomes, such as preterm birth, may be explained by increased maternal pregnancy risks and complications.

In the context of these gaps in the literature, our objective was to describe birth outcomes of infants born to mothers with IDD and compare them to infants born to mothers without IDD in a cohort of live births to mothers covered by Wisconsin Medicaid. Data were from the Big Data for Little Kids project (BD4LK) that links Medicaid claims and birth record data for infants born

between 2007 and 2016. We examined results adjusting for demographic factors and maternal pregnancy complications and grouping by IDD-type. We hypothesized that odds of preterm birth and being small for gestational age would be increased for infants born to mothers with IDD compared to those born to mothers without IDD, even after adjusting for demographic and maternal pregnancy factors.

Methods

BD4LK and sample derivation

We used data from BD4LK, a longitudinal cohort of Wisconsin birth records for in state live births from 2007-2016. Live birth records were linked to administrative data sources including claims and encounters in the Wisconsin Medicaid system. Birth/death record data are linked to maternal Medicaid demographic files by deterministic matching by full name and birth date. This file is linked to claims by unique Medicaid ID. BD4LK has access to claims from up to one-year predelivery to delivery for all Medicaid deliveries (bounded by January 1, 2007 and December 31, 2016). In 2011, Wisconsin transitioned from the 1999 Revision of the US Standard Certificate of Live Birth (2007-2010 records) to the 2003 Revision (2011-2016 records). Variables were harmonized across birth records although there are certain variables unique to the 2003 revision that are only available in 2011-2016 births (i.e. breastfed at discharge, maternal BMI). Additional detail on BD4LK can be found elsewhere (12-14).

Our sample was comprised of all deliveries to mothers with at ≥1 Medicaid-paid delivery in Wisconsin during 2007-2016. Starting with 666,375 birth records in BD4LK, we excluded

deliveries to mothers who never linked to a Medicaid claim for live delivery (N=381,879; 57.3%) or with imperfect linkages across Medicaid claims (N=1,825; <0.3%). We excluded deliveries to mothers who's only observed Medicaid-paid delivery occurred in 2013 due to potential data missingness related to data availability from Medicaid (N=7,806; 1.2%). Thus, our final analytic sample was of 274,865 infants (41.2% of all BD4LK birth records) born to 177,697 mothers.

Due to the time-bounds of our Medicaid data and the lack of availability of 2013 claims we created a subsample excluding births in 2007, 2013, and 2014, which ensured all mothers had the potential to have up to one year of prepregnancy claims. We used this sample of N = 195,691 children (71.2% of full analytic sample) in sensitivity analyses.

Classifying maternal IDD

Maternal IDD was determined by assessing Medicaid claims for International Classification of Disease 9 and 10 codes for IDD (15, 16). Women with IDD qualify for Medicaid by meeting income and asset requirements or by receiving a Social Security Disability Determination (which also has income requirements). IDD codes were identified from previous literature (4, 17-19) and are presented in Supplement 1. We grouped IDD into categories to examine effects of specific conditions: intellectual disability, genetic or chromosomal anomalies that cause IDD ('genetic conditions'), cerebral palsy, and autism spectrum disorder. Mothers could be in more than one category.

Infant outcomes and covariates

Data for infant outcomes were from the birth record: clinical estimate of gestational age in completed weeks, categorical gestational age in weeks (<32, 32-34, 35-36, >37), preterm birth <37 weeks), birthweight and low birth weight (<2500 grams), <5th or <10th percentile size for gestational age (20), Apgar 5 minute score as a continuous variable and categorized as 1-3, 4-6, and 7-10 (21), infant transfer to other medical facility, infant death before 12 months of age, neonatal intensive care unit (NICU) admission (2011-2016 births only, N=156,135), and being breast fed at discharge (2011-2016 births only, N=156,135/

Demographic covariates from the birth record included birth year, birth order, plurality, child sex, urban rural classification of birth county (derived from National Center for Health Statistics 2013 Urban Rural Classification Scheme; (22)), and maternal race, ethnicity, age, nativity, and education. We evaluated whether there was information on the father on the birth record (based on having a response for paternal age, education, or race) as infant health outcomes are worse for infants without father's information on the birth record compared to other children (23, 24).

Statistical analysis

We calculated distributions for categorical variables and means, standard deviations, median, and interquartile range for continuous variables. We ran multi-level logistic regression clustered by mother to calculate unadjusted odds ratios (uOR) and 95% confidence intervals (95% CI) and added additional adjustment sets to calculate adjusted odds ratios (aOR) for outcomes with adequate sample. First, we ran unadjusted analyses. Then, we added covariates for demographic characteristics: maternal race and Hispanic ethnicity, maternal age, urban-rural classification of birth county, father's information on birth record, and birth year. Then, we added maternal

healthcare use and pregnancy complications: prenatal care in the first trimester, gestational hypertension, gestational diabetes, induction, caesarean delivery, tobacco use during pregnancy, and parity. Lastly, we ran a fully adjusted model excluding plural births as plurality is associated with preterm birth and small for gestational age (25). We then assessed most common outcomes (preterm birth, size for gestational age, and low birth weight) by IDD type. For the IDD-types with adequate sample size (intellectual disability and genetic conditions) we also ran the model adjusting for the covariate sets described above. We ran analyses using SAS version 9.4 (SAS Institute, Cary, NC). This study was approved by the University of Wisconsin-Madison Institutional Review Board.

Results

Of 274,865 infants in our final analytic sample, 1757 were born to mothers with IDD (1032 unique mothers). Five-hundred fifty-five children were born to mothers with intellectual disability, 777 had a mother with a genetic condition, 279 had a mother with cerebral palsy, and 156 had a mother with autism spectrum disorder. Of those born to mothers with IDD, the maternal racial and ethnic distribution was 71.3% white, 21.0% black, and 10.6% were Hispanic (Table 1). Overall, 5.6% of the births to mothers with IDD were to foreign born mothers. . Of infants born to mothers without IDD (N=273,108), the maternal racial ethnic distribution was 72.0% white, 19.2% black and 14.6% were born to Hispanic mothers. Overall, 10.1% of the births to mothers without IDD were to foreign born.

The incidence of preterm birth was 14.9% in infants born to mothers with IDD compared to 9.2% among infants born to mothers without IDD (uOR: 1.60, 95% CI: 1.4, 1.8) (Table 2). The

incidence of very preterm birth (<32 weeks) in infants of mothers with IDD was two times that of infants of mothers without IDD (2.8% compared to 1.4%; uOR: 2.28, 95% CI: 1.6, 3.2). Of infants born to mothers with IDD, 14.3% were <2500 grams at birth compared to 8.1% of infants born to mothers without IDD (uOR: 1.90, 95% CI: 1.6, 2.2). When accounting for both gestational age and birth weight, infants born to mothers with IDD had higher odds of being in the 5th percentile or lower for size for gestational age (8.5% compared to 5.4%; uOR: 1.62, 95% CI: 1.3, 2.0) and 10th percentile or lower (15.5% compared to 11.1%; uOR: 1.47, 95% CI: 1.3, 1.7).

For post-natal outcomes, a greater percentage of infants born to mothers with IDD were transferred to another hospital (8.5%) or the NICU (14.6%) as compared to infants of mothers without IDD (3.9% and 8.7% respectively). Conversely, a lower percentage of infants of mothers with IDD were breastfed at discharge (57.7%) compared to infants of mothers without IDD (69.7%). Of note, infant death was reported for 62 children to mothers with IDD (3.5%) and 1959 infant deaths to mothers without IDD (0.7%). In both groups approximately 60% of the infants died within 28 days of birth.

We found little difference when adding demographic characteristics, maternal health care use and pregnancy complications, and restricting to singleton births (Figure 1, point estimates and 95% CI presented in Supplement 2). We additionally performed sensitivity analysis restricting our data to infants whose mothers had no pregnancy complications (N=70456, 26.6% of full sample) rather than adjusting for each condition separately: results did not differ (Supplement 2).

While there was some variance in estimates, results were consistent in direction and magnitude across models with little clear patterns of attenuation or strengthening with additional covariates.

Compared to infants born to mothers without IDD, the odds of preterm birth were higher for infants born to mothers of all IDD types (Table 3). Maternal intellectual disability, genetic conditions, and cerebral palsy were each associated with increased odds of being born <2500 grams. Both maternal intellectual disability and genetic conditions were associated with increased odds of being <5th percentile and <10th percentile size for gestational age. However, after adjustment for demographic characteristics and maternal complications, the association between size for gestational age and intellectual disability was attenuated and no longer statistically significant (Supplement 3).

Discussion

Infants of mothers with IDD are likely to be at increased risk of adverse birth outcomes due to a combination of biological and socioeconomic factors. Our goal was to characterized disparities in birth outcomes for infants born to mothers with IDD, an understudied but growing population for which more research is vitally needed (6, 18). We compared birth outcomes by maternal IDD status within the population of infants whose mothers had Medicaid-covered deliveries, examining the impact of maternal demographics, healthcare use, and pregnancy complications. Across IDD-types and adjustment sets, infants born to mothers with IDD had higher odds of preterm birth, low birth weight, being small for gestational age, NICU admission, and infant death, as well as lower odds of being breastfed at discharge.

Our findings from a Wisconsin sample are consistent with past research on infant birth outcomes of mothers with IDD. Using the National Inpatient Sample, researchers found 13% of infant of mothers with IDD were born preterm compared to 8% of those born to mothers without IDD (4, 11). In California linked hospital discharge records from 2000-2010, 15.1% of infants born to mothers with IDD were preterm, significantly greater than the full cohort (26). In a similar linkage in Massachusetts, 12.4% of infants born to mothers with IDD were low birth weight compared to 5.4% in the non-IDD sample (27). In the Ontario health care system, 9.8% of infants born to mothers with IDD were preterm and 17.2% were <10th percentile for gestational age (10). Our study reaffirms past findings on outcomes for infants of mothers with IDD and adds estimates for additional outcomes such as Apgar scores, and being breastfed at discharge. While our data were from a health insurance plan in Wisconsin designed for low-income individuals, results were consistent with other samples of women with IDD in the US and Canada that were not restricted to one health plan.

We found five-times the odds of infant death for infants born to mothers with IDD compared to infants born to mothers without IDD, a finding similar to a study done of women with intellectual disability in Sweden (28). The increased risk of maternal complications, poor infant outcomes, and other infant health conditions associated with maternal IDD could result in failure to thrive (29) or birth defects (30) and possibly drive increased infant mortality. Women with IDD are capable of parenting and raising families (31), and our findings should not be interpreted otherwise. With additional years of data and further data on cause of death, we will be better able to understand pathways leading to increased mortality risk.

Demographic factors such as race (as a proxy for racism and disparity) and education are associated with birth complications (32-34) and a larger percentage of mothers with IDD in our data are black and have less education then mothers without IDD. Yet, compared to a general population comparison group the Medicaid-based sample comparison group is more similar to IDD-group in respect to demographic factors. The general similarity between the mothers with and without IDD may explain the minimal change we saw when adjusting for maternal race, ethnicity, age, geographic county, and education. We relied on the birth record for demographic variables and more detailed phenotypic and health history data are needed. In our data, mothers with IDD were at greater risk of gestational diabetes, gestational hypertension, and caesarean delivery compared to mothers without IDD; however, adjustment for these factors or restriction to infants of mothers without any complication made little impact on estimates for birth outcomes for infants. Maternal health care use and pregnancy complications may act as mediators on the pathway from maternal IDD to birth outcomes (35, 36), and while that was not manifest in our analysis that explored the direct effect of maternal IDD on outcomes, a more formal mediation analysis with data that can meet the assumptions of no unmeasured confounding (37) may be needed.

We saw increased odds of preterm birth, low birth weight, and being small for gestational age among infants of mothers with intellectual disability, genetic conditions, and cerebral palsy compared to mothers without IDD. Effects were smaller for autism spectrum disorder, which needs to be further explored with a larger sample. Our findings for infants of mothers with intellectual disability or cerebral palsy were aligned with past work on those specific conditions (38, 39). Similar outcomes may indicate similar social and health disparities faced across IDD

types. Still, within IDD type and across all IDD there is heterogeneity in presentation, co-occurring conditions, and disparities faced (40). These data are conditioned on live birth, so differential fertility (41) or pregnancy loss (38) by IDD-type are needed to understand the full picture of pregnancy. More detailed phenotypic and lifestyle to describe women with IDD can help inform outcomes specific to condition. With larger cohorts young women with IDD, specifically autism spectrum disorder (42), entering reproductive age, the power to identify specific mechanisms and effects within IDD types might be increased.

With elevated odds across adjustment sets and IDD-type, our findings raise questions as to why infants of women with IDD have elevated risk. Some outcomes, like NICU admission or infant transfer, may be a result of preterm birth (43). Residual confounding is likely a factor, as we only had information from the birth record which may not capture the extent of demographic differences between mothers with and without IDD. Women with IDD often lack access to appropriate sexual and reproductive healthcare (2, 6, 44) which can directly impact health behavior surrounding pregnancy and result in poor birth outcomes (45, 46). More data on pregnancy planning and pregnancy-related health-service usage in women with IDD are needed to explore this potential mechanism. Compared to women without IDD, women with IDD are also at greater risk of having co-occurring conditions such as anxiety, depression, epilepsy, and cardiovascular disease (19) and may be taking medication for those indications. These health conditions and their medication may increase risk for poor birth outcomes (47, 48). It will be important to further explore these factors so we can best serve women with IDD and their infants through pregnancy and infancy.

Our study was limited by our reliance on Medicaid claims for identifying IDD. We relied on prepregnancy claims over a limited time period and some mothers may have only entered the Medicaid system later in pregnancy or at childbirth. As other studies have used inpatient hospitalization to determine IDD, we believe our claims data captures a window in which IDD can be identified. Medicaid data are conditioned on being low-income; replication in an all payer claims database may expand findings outside the low-income population. We were limited to assessing outcomes and covariates from the birth record which may underreport maternal complications (49). In only having demographic data from the birth record, we did not have characteristics like living arrangement, employment status, and pregnancy planning which could inform findings. Our results are representative of Wisconsin Medicaid form 2007-2016 and may not be generalizable to other time frames or other populations.

Conclusion

We found that among Medicaid covered births, infants born to mothers with IDD were at elevated odds of preterm birth, low birth weight, being small for gestational age, being transferred to the NICU, and infant death compared to children of mothers without IDD. Our findings were robust to adjustment for demographic factors, maternal health care use and pregnancy complications, and results were similar IDD-type. It is imperative to understand why children of mothers with IDD are at elevated risk so interventions and management can be developed to improve outcomes in the future.

The authors of this article are solely responsible for the content therein. The authors would like to thank the Department of Health Services, for the use of data for this analysis, but these agencies do not certify the accuracy of the analyses presented.

References

1. Durkin M, Rubenstein E. Epidemiology of intellectual and developmental disabilties Glidde L, editor: Springer; 2020 In press
2. Greenwood NW, Wilkinson J. Sexual and reproductive health care for women with intellectual disabilities: a primary care perspective. Int J Family Med. 2013;2013:642472. doi: 10.1155/2013/642472. PubMed PMID: 24455249; PMCID: 3876698.
3. Roy A, Roy A, Roy M. The human rights of women with intellectual disability. J R Soc Med. 2012;105(9):384-9. doi: 10.1258/jrsm.2012.110303. PubMed PMID: 22977048; PMCID: 3439660.
4. Parish SL, Mitra M, Son E, Bonardi A, Swoboda PT, Igdalsky L. Pregnancy Outcomes Among U.S. Women With Intellectual and Developmental Disabilities. Am J Intellect Dev Disabil. 2015;120(5):433-43. doi: 10.1352/1944-7558-120.5.433. PubMed PMID: 26322390.
5. McConnell D, Mayes R, Llewellyn G. Women with intellectual disability at risk of adverse pregnancy and birth outcomes. J Intellect Disabil Res. 2008;52(Pt 6):529-35. doi: 10.1111/j.1365-2788.2008.01061.x. PubMed PMID: 18422528.
6. Mueller BA, Crane D, Doody DR, Stuart SN, Schiff MA. Pregnancy course, infant outcomes, rehospitalization, and mortality among women with intellectual disability. Disabil Health J. 2019;12(3):452-9. Epub 2019/01/30. doi: 10.1016/j.dhjo.2019.01.004. PubMed PMID: 30692054; PMCID: PMC6581578.
7. Altman BM, Madans J, Weeks JD. An evaluation of the American Community Survey indicators of disability. Disabil Health J. 2017;10(4):485-91. Epub 2017/04/08. doi: 10.1016/j.dhjo.2017.03.002. PubMed PMID: 28385572.
8. Graham H. Intellectual Disabilities and Socioeconomic Inequalities in Health: An Overview of Research. Journal of Applied Research in Intellectual Disabilities. 2005;18(2):101-11. doi: 10.1111/j.1468-3148.2005.00239.x.
9. Krahn GL, Fox MH. Health disparities of adults with intellectual disabilities: what do we know? What do we do? J Appl Res Intellect Disabil. 2014;27(5):431-46. doi: 10.1111/jar.12067. PubMed PMID: 23913632; PMCID: 4475843.
10. Brown HK, Cobigo V, Lunsky Y, Dennis CL, Vigod S. Perinatal Health of Women with Intellectual and Developmental Disabilities and Comorbid Mental Illness. Can J Psychiatry. 2016;61(11):714-23. doi: 10.1177/0706743716649188. PubMed PMID: 27310242; PMCID: 5066551.
11. Akobirshoev I, Parish SL, Mitra M, Rosenthal E. Birth outcomes among US women with intellectual and developmental disabilities. Disabil Health J. 2017;10(3):406-12. doi: 10.1016/j.dhjo.2017.02.010. PubMed PMID: 28404230; PMCID: 5477666.
12. Larson A, Berger LM, Mallinson DC, Grodsky E, Ehrenthal DB. Variable Uptake of Medicaid-Covered Prenatal Care Coordination: The Relevance of Treatment Level and Service Context. J Community Health. 2019;44(1):32-43. Epub 2018/07/20. doi: 10.1007/s10900-018-0550-9. PubMed PMID: 30022418; PMCID: PMC6330123.
13. Mallinson DC, Ehrenthal DB. Classification of Medicaid Coverage on Birth Records in Wisconsin, 2011-2012. Public Health Rep. 2019;134(5):542-51. Epub 2019/07/04. doi: 10.1177/0033354919860503. PubMed PMID: 31269411; PMCID: PMC6852064.

14. Mallinson DC, Larson A, Berger LM, Grodsky E, Ehrenthal DB. Estimating the effect of Prenatal Care Coordination in Wisconsin: A sibling fixed effects analysis. Health Serv Res. 2020;55(1):82-93. Epub 2019/11/09. doi: 10.1111/1475-6773.13239. PubMed PMID: 31701531; PMCID: PMC6980950.
15. World Health Organization. ICD-10 : international statistical classification of diseases and related health problems : tenth revision. 2nd ed ed. Geneva: World Health Organization; 2004.
16. World Health Organization. International classification of diseases : [9th] ninth revision, basic tabulation list with alphabetic index. Geneva: World Health Organization; 1978.
17. McDermott S, Royer J, Cope T, Lindgren S, Momany E, Lee JC, McDuffie MJ, Lauer E, Kurtz S, Armour BS. Using Medicaid Data to Characterize Persons With Intellectual and Developmental Disabilities in Five U.S. States. Am J Intellect Dev Disabil. 2018;123(4):371-81. Epub 2018/06/28. doi: 10.1352/1944-7558-123.4.371. PubMed PMID: 29949427.
18. Brown HK, Chen S, Guttmann A, Havercamp SM, Parish S, Ray JG, Tarasoff LA, Vigod SN, Carty A, Lunsky Y. Rates of recognized pregnancy in women with disabilities in Ontario, Canada. Am J Obstet Gynecol. 2020;222(2):189-92. Epub 2019/11/07. doi: 10.1016/j.ajog.2019.10.096. PubMed PMID: 31689381.
19. Bishop-Fitzpatrick L, Rubenstein E. The Physical and Mental Health of Middle Aged and Older Adults on the Autism Spectrum and the Impact of Intellectual Disability. Res Autism Spectr Disord. 2019;63:34-41. Epub 2019/11/27. doi: 10.1016/j.rasd.2019.01.001. PubMed PMID: 31768189; PMCID: PMC6876625.
20. Talge NM, Mudd LM, Sikorskii A, Basso O. United States birth weight reference corrected for implausible gestational age estimates. Pediatrics. 2014;133(5):844-53. Epub 2014/04/30. doi: 10.1542/peds.2013-3285. PubMed PMID: 24777216.
21. Committee Opinion No. 644: The Apgar Score. Obstet Gynecol. 2015;126(4):e52-5. Epub 2015/09/24. doi: 10.1097/aog.0000000000001108. PubMed PMID: 26393460.
22. Ingram DD, Franco SJ. 2013 NCHS urban–rural classification scheme for counties. . In: Statistics NCfH, editor.: Vital Health Statistics 2014.
23. Almond D, Rossin-Slater M. Paternity acknowledgment in 2 million birth records from Michigan. PLoS One. 2013;8(7):e70042. Epub 2013/07/31. doi: 10.1371/journal.pone.0070042. PubMed PMID: 23894583; PMCID: PMC3718738.
24. Cheng ER, Hawkins SS, Rifas-Shiman SL, Gillman MW, Taveras EM. Association of missing paternal demographics on infant birth certificates with perinatal risk factors for childhood obesity. BMC Public Health. 2016;16:453. Epub 2016/07/15. doi: 10.1186/s12889-016-3110-1. PubMed PMID: 27411308; PMCID: PMC4944478.
25. Martin JA, Hamilton BE, Osterman M, Driscoll A. Births: Final data for 2018.
. Hyattsville, MD: 2019.
26. Darney BG, Biel FM, Quigley BP, Caughey AB, Horner-Johnson W. Primary Cesarean Delivery Patterns among Women with Physical, Sensory, or Intellectual Disabilities. Womens Health Issues. 2017;27(3):336-44. Epub 2017/01/23. doi: 10.1016/j.whi.2016.12.007. PubMed PMID: 28109562; PMCID: PMC5435518.
27. Mitra M, Parish SL, Akobirshoev I, Rosenthal E, Moore Simas TA. Postpartum Hospital Utilization among Massachusetts Women with Intellectual and Developmental Disabilities: A

Retrospective Cohort Study. Matern Child Health J. 2018;22(10):1492-501. Epub 2018/06/28. doi: 10.1007/s10995-018-2546-6. PubMed PMID: 29948759; PMCID: PMC6150791.
28. Hoglund B, Lindgren P, Larsson M. Newborns of mothers with intellectual disability have a higher risk of perinatal death and being small for gestational age. Acta Obstet Gynecol Scand. 2012;91(12):1409-14. Epub 2012/08/29. doi: 10.1111/j.1600-0412.2012.01537.x. PubMed PMID: 22924821; PMCID: PMC3549565.
29. Cassidy SB, Schwartz S, Miller JL, Driscoll DJ. Prader-Willi syndrome. Genet Med. 2012;14(1):10-26. Epub 2012/01/13. doi: 10.1038/gim.0b013e31822bead0. PubMed PMID: 22237428.
30. Auger N, Arbour L, Schnitzer ME, Healy-Profitos J, Nadeau G, Fraser WD. Pregnancy outcomes of women with spina bifida. Disabil Rehabil. 2019;41(12):1403-9. Epub 2018/01/13. doi: 10.1080/09638288.2018.1425920. PubMed PMID: 29327608.
31. McConnell D, Feldman M, Aunos M. Parents and parenting with intellectual disabilities: An expanding field of research. J Appl Res Intellect Disabil. 2017;30(3):419-22. Epub 2017/04/12. doi: 10.1111/jar.12362. PubMed PMID: 28397394.
32. Bryant AS, Worjoloh A, Caughey AB, Washington AE. Racial/ethnic disparities in obstetric outcomes and care: prevalence and determinants. Am J Obstet Gynecol. 2010;202(4):335-43. Epub 2010/01/12. doi: 10.1016/j.ajog.2009.10.864. PubMed PMID: 20060513; PMCID: PMC2847630.
33. Gage TB, Fang F, O'Neill E, Dirienzo G. Maternal education, birth weight, and infant mortality in the United States. Demography. 2013;50(2):615-35. Epub 2012/10/18. doi: 10.1007/s13524-012-0148-2. PubMed PMID: 23073749; PMCID: PMC3578151.
34. Carolan M, Frankowska D. Advanced maternal age and adverse perinatal outcome: a review of the evidence. Midwifery. 2011;27(6):793-801. doi: 10.1016/j.midw.2010.07.006. PubMed PMID: 20888095.
35. Ornoy A. Growth and neurodevelopmental outcome of children born to mothers with pregestational and gestational diabetes. Pediatric endocrinology reviews : PER. 2005;3(2):104-13. PubMed PMID: 16361984.
36. Berhe AK, Ilesanmi AO, Aimakhu CO, Mulugeta A. Effect of pregnancy induced hypertension on adverse perinatal outcomes in Tigray regional state, Ethiopia: a prospective cohort study. BMC Pregnancy Childbirth. 2019;20(1):7. Epub 2020/01/02. doi: 10.1186/s12884-019-2708-6. PubMed PMID: 31892353; PMCID: PMC6938605.
37. Valeri L, Vanderweele TJ. Mediation analysis allowing for exposure-mediator interactions and causal interpretation: theoretical assumptions and implementation with SAS and SPSS macros. Psychol Methods. 2013;18(2):137-50. Epub 2013/02/06. doi: 10.1037/a0031034. PubMed PMID: 23379553; PMCID: PMC3659198.
38. Goldacre AD, Gray R, Goldacre MJ. Childbirth in women with intellectual disability: characteristics of their pregnancies and outcomes in an archived epidemiological dataset. J Intellect Disabil Res. 2015;59(7):653-63. Epub 2014/10/22. doi: 10.1111/jir.12169. PubMed PMID: 25331275.
39. Sundelin HEK, Stephansson O, Johansson S, Ludvigsson JF. Pregnancy outcome in women with cerebral palsy: A nationwide population-based cohort study. Acta Obstet Gynecol Scand. 2020;99(4):518-24. Epub 2019/11/19. doi: 10.1111/aogs.13773. PubMed PMID: 31738455.

40. American Psychiatric Association. Diagnostic and Statistical Manual of Mental Disorders (5th ed.). Arlington, VA: American Psychiatric Publishing; 2013.
41. Elkins TE, McNeeley SG, Punch M, Kope S, Heaton C. Reproductive health concerns in Down syndrome. A report of eight cases. J Reprod Med. 1990;35(7):745-50. Epub 1990/07/01. PubMed PMID: 2142964.
42. Rubenstein E, Bishop L. Is the Autism Boom Headed for Medicaid? Patterns in the Enrollment of Autistic Adults in Wisconsin Medicaid, 2008-2018. Autism Res. 2019;12(10):1541-50. Epub 2019/07/19. doi: 10.1002/aur.2173. PubMed PMID: 31317639; PMCID: PMC7006836.
43. Ziegler KA, Paul DA, Hoffman M, Locke R. Variation in NICU Admission Rates Without Identifiable Cause. Hosp Pediatr. 2016;6(5):255-60. Epub 2016/04/28. doi: 10.1542/hpeds.2015-0058. PubMed PMID: 27117951.
44. Barnard-Brak L, Schmidt M, Chesnut S, Wei T, Richman D. Predictors of access to sex education for children with intellectual disabilities in public schools. Intellect Dev Disabil. 2014;52(2):85-97. Epub 2014/04/15. doi: 10.1352/1934-9556-52.2.85. PubMed PMID: 24725108.
45. Lang AY, Boyle JA, Fitzgerald GL, Teede H, Mazza D, Moran LJ, Harrison C. Optimizing preconception health in women of reproductive age. Minerva Ginecol. 2018;70(1):99-119. Epub 2017/09/13. doi: 10.23736/s0026-4784.17.04140-5. PubMed PMID: 28895680.
46. Stephenson J, Heslehurst N, Hall J, Schoenaker DAJM, Hutchinson J, Cade JE, Poston L, Barrett G, Crozier SR, Barker M, Kumaran K, Yajnik CS, Baird J, Mishra GD. Before the beginning: nutrition and lifestyle in the preconception period and its importance for future health. The Lancet. 2018;391(10132):1830-41. doi: 10.1016/s0140-6736(18)30311-8.
47. Razaz N, Tomson T, Wikström A-K, Cnattingius S. Association Between Pregnancy and Perinatal Outcomes Among Women With Epilepsy. JAMA Neurology. 2017;74(8):983-91. doi: 10.1001/jamaneurol.2017.1310.
48. Becker M, Weinberger T, Chandy A, Schmukler S. Depression During Pregnancy and Postpartum. Curr Psychiatry Rep. 2016;18(3):32. Epub 2016/02/18. doi: 10.1007/s11920-016-0664-7. PubMed PMID: 26879925.
49. Andrade SE, Scott PE, Davis RL, Li DK, Getahun D, Cheetham TC, Raebel MA, Toh S, Dublin S, Pawloski PA, Hammad TA, Beaton SJ, Smith DH, Dashevsky I, Haffenreffer K, Cooper WO. Validity of health plan and birth certificate data for pregnancy research. Pharmacoepidemiol Drug Saf. 2013;22(1):7-15. Epub 2012/07/04. doi: 10.1002/pds.3319. PubMed PMID: 22753079; PMCID: PMC3492503.

Table 1 Demographic characteristics of infants born to women with a Medicaid live birth delivery in Wisconsin, 2007-2016; by intellectual and developmental disability status

	Infants of mothers with intellectual and developmental disability N=1757		Infants of mothers without intellectual and developmental disabilities N=273108	
	N	%	N	%
**Year of birth**				
2007	180	10.2	28375	10.4
2008	243	13.8	29032	10.6
2009	222	12.6	30153	11.0
2010	192	10.9	29242	10.7
2011	195	11.1	28983	10.6
2012	193	11.0	28840	10.6
2013	136	7.7	25242	9.2
2014	149	8.5	25092	9.2
2015	127	7.2	24328	8.9
2016	120	6.8	23821	8.7
**Birth order**				
First born	557	31.7	93882	34.4
Second born	497	28.3	81326	29.8
Third born	334	19.0	51084	18.7
Fourth born or later	369	21.0	46717	17.1
Missing			99	
**Plurality**				
Multiple	61	3.5	7409	2.7
Singleton	1696	96.5	265699	97.3
**Child sex**				
Male	912	51.9	139561	51.1
Female	845	48.1	133545	48.9
**County size where child was born** [a]				
Large central metro	546	31.1	78540	28.8
Large fringe metro	135	7.7	25553	9.4
Medium metro	247	14.1	39540	14.5
Small metro	448	25.5	70671	25.9
Micropolitan	195	11.1	32149	11.8
Non-core	186	10.6	26655	9.8
**Maternal age at childbirth**				
<=18	68	3.9	8919	3.3
19-24	772	43.9	118677	43.5
25-29	486	27.7	81042	29.7
30-34	264	15.0	43932	16.1
35-39	123	7.0	16847	6.2
>=40	44	2.5	3691	1.4
**Maternal race**				
White	736	71.3	196684	72.0
Black	223	21.6	52398	19.2
Asian	49	4.7	17246	6.3
Other	24	2.3	6780	2.5
**Hispanic ethnicity**				
Hispanic	187	10.6	39819	14.6

Non-Hispanic	1570	89.4	233288	85.4
**Mother foreign nativity**				
Yes	99	5.6	27614	10.1
No	1658	94.4	245489	89.9
Missing	-		-	
**Maternal education**				
<High school	473	27.2	59093	21.6
Completed high school	826	47.4	111017	40.6
Some college	349	20.0	80407	29.4
>= Completed college	93	5.3	20801	7.6
Missing	16		1706	
**Father presence on birth record** [b]				
Yes	1521	86.6	248063	90.8
No	236	13.4	25045	9.2
**Caesarean delivery**				
Yes	436	26.3	54366	20.8
No	1220	73.7	206608	79.2
**Induced delivery**				
Yes	498	28.4	74963	27.5
No	1254	71.6	197925	72.5
**Tobacco use during pregnancy**				
Yes	521	29.9	72412	26.6
No	1220	70.1	199441	73.4
**Gestational diabetes**				
Yes	123	7.0	14985	5.5
No	1634	93.0	258123	94.5
**Gestational hypertension**				
Yes	106	6.0	13744	5.0
No	1651	94.0	259364	95.0
**Prenatal care**				
Yes	1241	72.8	194549	73.4
No	463	27.2	70490	26.6

Cells with N<10 are suppressed

[a] Based on National Center for Health Statistics 2013 Urban Rural Classification Scheme

[b] Father presence on birth record determined by reported paternal age

Table 2 Occurrence and odds ratios of birth outcomes comparing infants born to mothers with and without intellectual and developmental disabilities in Wisconsin Medicaid, 2007-2016

	Infants born to mothers with Intellectual and developmental disabilities N=1757		Infants born to mothers without intellectual and developmental disabilities N=273108		Unadjusted odds ratio[a]	
	N	%	N	%	OR	95% CI
**Gestational age**						
Preterm	261	14.9	25037	9.2	**1.60**	1.4, 1.8
Preterm category						
<32 weeks	49	2.8	3819	1.4	**2.28**	1.6, 3.2
32-34 weeks	61	3.5	6357	2.3	1.32	0.9, 1.9
35-36 weeks	151	8.6	148561	5.5	**1.54**	1.4, 1.8
Term	1488	85.1	247358	90.8	REF	
Missing	-		721			
Mean, SD (weeks)	38.1	2.6	38.6	2.0		
Median, IQR	39.0	2.0	39.0	2.0		
**Birth weight**						
<2500 grams	251	14.3	22165	8.1	**1.90**	1.6, 2.2
≥2500 grams	1501	85.6	250804	91.9	REF	
Missing	-		137			
Mean, SD (grams)	3110.7	675.8	3263.3	589.8		
Median, IQR	3175.0	723.0	3289.0	681.0		
**Size for gestational age**						
≤5th percentile	148	8.5	14912	5.4	**1.62**	1.3, 2.0
≤10th percentile	271	15.5	30348	11.1	**1.47**	1.3, 1.7
>10th percentile	1477	84.5	242047	88.9	REF	
Missing	-		729			
**Apgar 5-minute score**						
1-3	29	1.7	1359	0.5	**3.36**	2.3, 5.0
4-6	44	2.5	4178	1.5	**1.66**	1.2, 2.3
7-10	1680	95.8	266858	98.0	REF	
Missing	-		1248			
	8.63	1.2	8.84	0.8		

Mean, SD						
Median, IQR	9.0	0	9.0	0		
**Transfer to other medical facility**						
Yes	144	8.5	10625	3.9	**2.48**	2.1, 3.0
No	1550	91.5	260126	95.5	REF	
Missing	-		398			
**NICU admission**[b]						
Yes	133	14.6	13723	8.7	**1.79**	1.5, 2.2
No	781	85.5	142354	91.2	REF	
Missing	-		229			
**Breastfed at discharge**[b]						
Yes	479	57.7	99745	69.7	**0.57**	0.5, 0.7
No	351	42.3	43416	30.3	REF	
Missing	90		13145			
**Infant death**						
Yes	62	3.5	1959	0.7	**5.14**	3.9, 6.7
No	1657	96.5	271149	99.3	REF	

SD: standard deviation, IQR: interquartile range, NICU: neonatal intensive care unit, CI: confidence interval, REF: reference
[a] Unadjusted odds ratios clustered by mother
[b] NICU admission and breast fed at discharge only for years 2011-2016; N=155252
Bold indicates statistical significance at an alpha=0.05 level.
Cells with values <10 are suppressed

Figure 1 Odds ratios for prevalent infant birth outcomes comparing children born to mothers with and without intellectual disabilities in Wisconsin Medicaid, 2007-2016

Demographics: maternal race, categorized age, Hispanic ethnicity, geographic county size of birth county, father's information on birth record, birth year
Maternal health care use and pregnancy complications: Prenatal care in the first trimester, gestational hypertension, gestational diabetes, caesarean delivery, induction, parity, tobacco use during pregnancy
NICU admission and breastfed at discharge for 2011-2016 births only
All odds ratios clustered by mother

Table 3 Occurrence and odds ratios for birth outcomes comparing infants born to mothers with and without intellectual and developmental disabilities in Wisconsin Medicaid 2007-2016, by intellectual and developmental disability subtype

	Intellectual disability N=552				Genetic condition N=777				Cerebral Palsy N=279				Autism N=156			
	N	%	OR	95% CI	N	%	OR	95% CI	N	%	OR	95% CI	N	%	OR	95% CI
**Gestational age**																
Preterm	85	15.5	**1.78**	1.4, 2.3	111	14.3	**1.70**	1.4, 2.1	51	18.5	**2.06**	1.4, 3.0	23	14.9	**1.72**	1.1, 2.8
Term	464	84.5			666	85.7			225	81.5			131	85.1		
Mean, SD	38.2	2.5			38.1	2.3			37.8	3.2			38.4	2.2		
Median, IQR	39.0	3.0			39.0	2.0			39.0	2.0			39.0	2.0		
**Birth weight**																
<2500 grams	77	14.0	**1.87**	1.4, 2.5	117	15.1	**2.05**	1.6, 2.6	43	15.5	**1.95**	1.3, 2.9	17	11.1	1.36	0.8, 2.4
>=2500 grams	475	86.0			660	84.9			234	84.5			136	88.9		
Mean, SD	3089.9	681.3			3134.6	666.6			3039.9	732.8			3209.8	563.4		
Median, IQR	3118.0	716.5			3430.0	744.0			3119.0	707.0			3260.0	656.0		
**Size for gestational age**																
≤5th percentile	50	9.1	**1.81**	1.3, 2.4	71	9.1	**1.74**	1.3, 2.3	18	6.5	1.20	0.7, 2.1	11	7.2	1.31	0.6, 2.7
≤10th percentile	92	16.8	**1.64**	1.3, 2.1	114	14.7	**1.39**	1.1, 1.7	45	16.3	**1.52**	1.0, 2.2	22	14.4	1.32	0.8, 2.2
>10th percentile	457	83.2			663	85.2			231	83.7			131	85.6		

SD: standard deviation, IQR: interquartile range; OR: odds ratio; CI: confidence interval
Missing values suppressed since cells <10
Odds ratios clustered by mother, unadjusted

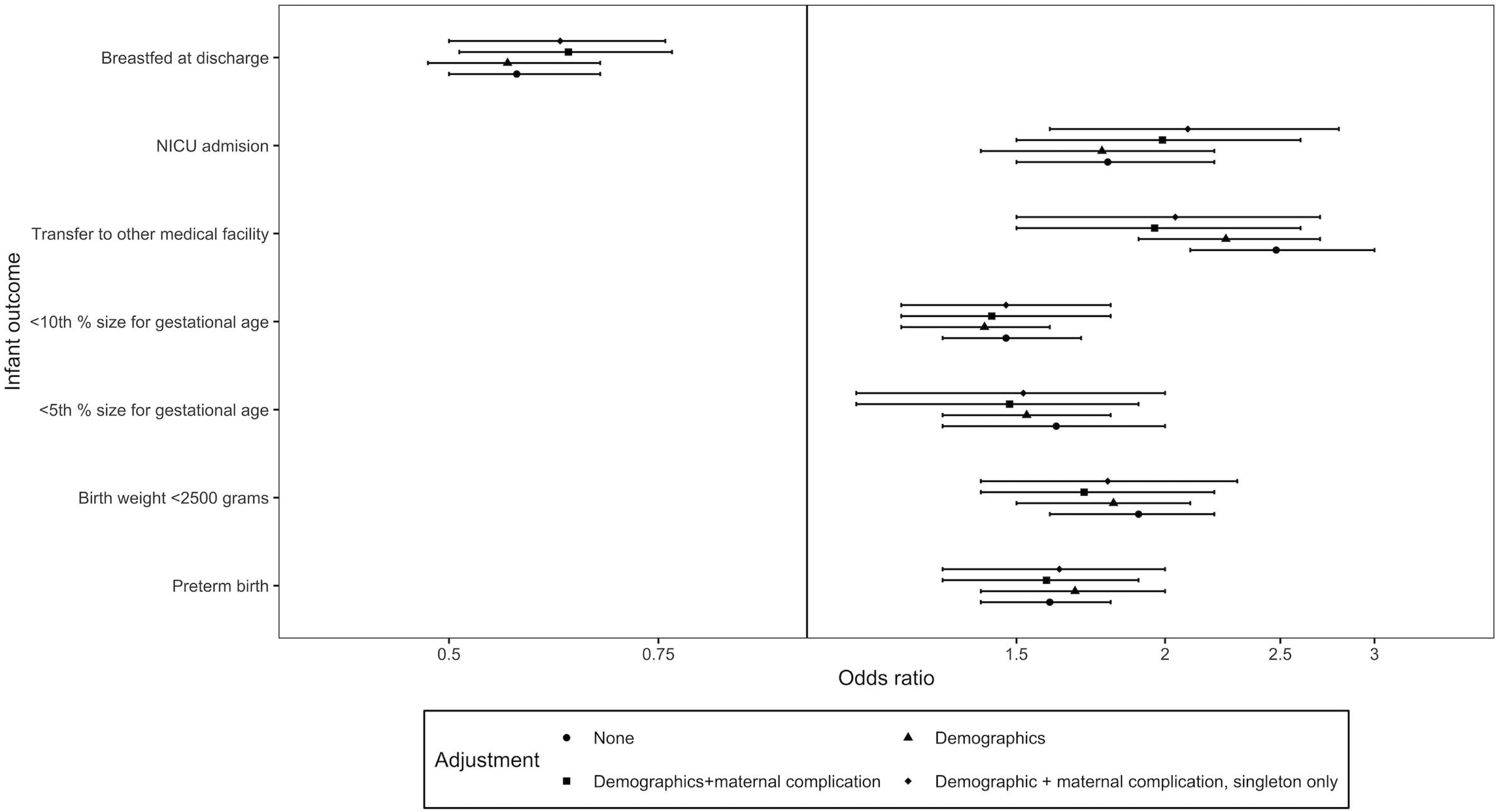

Breastfed at discharge
NICU admision
Transfer to other medical facility
<10th % size for gestational age
<5th % size for gestational age
Birth weight <2500 grams
Preterm birth
Infant outcome
0.5
0.75
1.5
2
2.5
3
Odds ratio
Adjustment
None
Demographics
Demographics+maternal complication
Demographic + maternal complication, singleton only
